# Comorbidity of autism with hyperkinetic disorder

**DOI:** 10.1192/j.eurpsy.2021.591

**Published:** 2021-08-13

**Authors:** A. Koval-Zaytsev, N. Simashkova, M. Ivanov

**Affiliations:** Department Of Child Psychiatry, Federal State Budgetary Scientific Institution “Mental Health Research Center”, Moscow, Russian Federation

**Keywords:** autism spectrum disorders, comorbidity, hyperkinetic disorder, psychodiagnostics

## Abstract

**Introduction:**

Autism spectrum disorders encompass a heterogeneous group of neurodevelopmental disorders. Autism may be accompanied by other mental and neurological disorders. Comorbidity in autism is the rule rather than the exception (as reflected in DSM-5).

**Objectives:**

To study comorbidity in patients with childhood autism and hyperkinetic disorder.

**Methods:**

Surveyed 102 patients aged 6–7 years who had infantile psychosis before the age of 3 years (F84.02), comorbid with hyperkinetic disorder (F90.0). Methods: clinical, psychological and psychometric (CARS, PEP, bfcrs, CGI, CPRS-R:S (parents’ form)).

**Results:**

In the surveyed patients, the autism level was 46 points according to CARS. Manifestations of hyperkinetic disorder in patients with F84.02 are found in 72%, which is associated with the severity of catatonic arousal (BFCRS 36 points). The cognitive development of the examined children is characterized by a combination of advancing, normative and delayed levels of development, depending on the type of cognitive dysontogenesis. Low indicators are revealed in involuntary attention, fine motor skills and hand-eye coordination. In patients with F84.02, a secondary hyperkinetic disorder forms upon exit from severe catatonia.

**Conclusions:**

Excessive motor activity is combined with impulsiveness and impaired attention in the period of remission. The use of a complex of clinical and psychodiagnostic techniques aimed at assessing voluntary and involuntary attention provides additional data for the diagnosis of ASD and hyperkinetic disorders.

